# Establishment of a human ovarian endometrioid carcinoma cell line by constitutive expression of cyclin-dependent kinase 4, cyclin D1 and telomerase reverse transcriptase

**DOI:** 10.1007/s13577-025-01176-0

**Published:** 2025-01-29

**Authors:** Hitomi Hoshino, Tomoya O. Akama, Daisuke Inoue, Suzuko Moritani, Shohei Shigeto, Kazuyuki Matsuda, Hisato Yoshida, Natsumi Yonemoto, Mana Fukushima, Yoshio Yoshida, Motohiro Kobayashi

**Affiliations:** 1 Department of Tumor Pathology, Faculty of Medical Sciences, University of Fukui, 23-3 Matsuoka-Shimoaizuki, Eiheiji, Fukui, 910-1193 Japan; 2 Department of Pharmacology, Kansai Medical University, Hirakata, Japan; 3 Department of Obstetrics and Gynecology, Faculty of Medical Sciences, University of Fukui, Eiheiji, Japan; 4 Division of Diagnostic Pathology, Shiga University of Medical Science Hospital, Otsu, Japan; 5 Department of Laboratory Medicine, Shinshu University Hospital, Matsumoto, Japan; 6 Department of Clinical Laboratory Sciences, School of Health Sciences, Shinshu University, Matsumoto, Japan; 7 Division of Surgical Pathology, University of Fukui Hospital, Eiheiji, Japan

**Keywords:** Ovary, Endometrioid carcinoma, Cyclin-dependent kinase 4, Cyclin D1, Telomerase reverse transcriptase

## Abstract

Only a few human ovarian endometrioid carcinoma cell lines are currently available, partly due to the difficulty of establishing cell lines from low-grade cancers. Here, using a cell immortalization strategy consisting of i) inactivation of the p16^INK4a^-pRb pathway by constitutive expression of mutant cyclin-dependent kinase 4 (R24C) (CDK4^R24C^) and cyclin D1, and ii) acquisition of telomerase reverse transcriptase (TERT) activity, we established a human ovarian endometrioid carcinoma cell line from a 46-year-old Japanese woman. That line, designated JFE-21, has proliferated continuously for over 6 months with a doubling time of ~ 55 h. JFE-21 cells exhibit polygonal shapes and proliferate without contact inhibition to form a monolayer in a jigsaw puzzle-like arrangement. Ultrastructurally, JFE-21 cells exhibit well-developed rough endoplasmic reticulum, mitochondria and lysosomes in the cytoplasm, with cells contacting each other via desmosomes. G-band karyotype analysis indicated that cells had a near-tetraploid karyotype. Immunofluorescence staining revealed that the expression profile of a series of ovarian carcinoma markers in JFE-21 cells was consistent with ovarian endometrioid carcinoma. Moreover, Sanger sequencing of DNA polymerase ε (*POLE*) gene and immunohistochemical analysis of mismatch repair (MMR) proteins revealed that JFE-21 cells were classified as the no specific molecular profile (NSMP) subtype. In addition, JFE-21 cells were sensitive to paclitaxel and carboplatin administered to the donor as therapy. These findings indicate that constitutive expression of CDK4^R24C^, cyclin D1 and TERT genes may be an option to establish cell lines from low-grade cancers, including ovarian endometrioid carcinoma.

## Introduction

More than 70 years have passed since the first human cancer cell line, HeLa cells, was established by George Otto Gey and colleagues in 1951 [1]. Since then, several human cancer cell lines have been established and are now extremely important tools for cancer research, contributing significantly to human health and welfare. On the other hand, paradoxically, cancer cells that proliferate autonomously in the human body often have limited ability to proliferate in vitro [2], making it extremely difficult to establish stable cell lines from many patient tumor samples. Thus, the success of cell line establishment is, in a sense, largely dependent on chance.

In the case of ovarian cancer, the difficulty of establishing human cell lines has been demonstrated in a comprehensive study by Verschraegen et al., who reported that of the 90 tumor samples, only 11 could be established as cell lines [3]. Furthermore, all cell lines established in that study were from high-grade serous or poorly differentiated carcinomas, and attempts to establish cell lines from low-grade endometrioid carcinomas have been unsuccessful [2]. Only a few ovarian endometrioid carcinoma cell lines are described in the literature [4–6]; thus, additional ovarian endometrioid carcinoma lines are needed to better understand the pathogenesis of this type of carcinoma and facilitate the development of effective treatments. One caveat is that cell lines with excessively long doubling times are not suitable for general experimental use [2].

The cell cycle is tightly regulated by several molecules, among which cyclin-dependent kinase 4 (CDK4) and cyclin D1 play a pivotal role in the transition from the G1 phase to S phase through phosphorylation of retinoblastoma protein (pRb). CDK4/cyclin D1-mediated pRb phosphorylation is negatively regulated by CDK inhibitor 2A (CDKN2A), also known as p16^INK4a^. p16^INK4a^ activation in response to cellular stress causes premature cell cycle arrest before telomere reduction. This cell growth suppression system is presumed to function in a cell culture environment [7]. To circumvent this inhibitory system and promote cell cycle progression, Shiomi et al. and Nishiwaki et al. induced constitutive expression of CDK4^R24C^, cyclin D1 and telomerase reverse transcriptase (TERT) in human myogenic cells isolated from healthy muscle [8] and human hepatocytes isolated from liver with biliary atresia [9], respectively. The amino acid substitution R24C in CDK4 protein renders its kinase activity resistant to inhibition by p16^INK4a^ [8], allowing cells to escape cell cycle arrest and continue proliferating.

In the present study, using the three-gene immortalization strategy described above, we established a human ovarian endometrioid carcinoma cell line from a 46-year-old Japanese woman. This line, designated JFE-21, has been proliferating continuously for more than 6 months with a doubling time of ~ 55 h, and exhibits expression profiles of a series of ovarian carcinoma markers, consistent with ovarian endometrioid carcinoma. Thus, constitutive expression of CDK4^R24C^, cyclin D1 and TERT genes may be an option for establishing cell lines from low-grade cancers, including ovarian endometrioid carcinoma.

## Materials and methods

### Patient’s clinical history

A 46-year-old Japanese woman, gravida 2, para 2, was referred to the hospital with complaints of dysmenorrhea. Systemic computed tomography (CT) and abdominal magnetic resonance imaging revealed a 6-cm-sized multilocular cystic tumor with solid lesions in the left ovary and a 5-cm-sized simple cystic tumor in the right ovary, along with a small amount of ascites. Serum cancer antigen 125 (CA125) and CA19-9 levels were 322 U/mL and 2,711 U/mL, respectively. These findings were suggestive of early-stage ovarian cancer with a background of endometriosis. A staging laparotomy including total abdominal hysterectomy, bilateral salpingo-oophorectomy, omentectomy and retroperitoneal lymphadenectomy was performed. Consequently, the patient was diagnosed with ovarian endometrioid carcinoma with a score of IC1, based on International Federation of Gynecology and Obstetrics (FIGO) staging. The patient was given six cycles of intravenous paclitaxel (175 mg/m^2^) and carboplatin (area under the concentration–time curve [AUC] of 6 mg·min/mL) on a 21-day cycle. Post-chemotherapy, systemic CT revealed complete response according to the Response Evaluation Criteria in Solid Tumors (RECIST version 1.1) [10]. The patient is currently living and has had no cancer recurrence for 40 months.

### Preparation of recombinant lentiviruses

Lentiviral vectors harboring cDNAs encoding human CDK4^R24C^ and cyclin D1 were constructed based on previous reports [8, 9]. Briefly, cDNA encoding CDK4 was amplified by polymerase chain reaction (PCR) and inserted into pcDNA3 (Thermo Fisher Scientific, Waltham, MA), and the R24C mutation was then introduced using inverse-PCR and *Dpn*I digestion (New England Biolabs, Ipswich, MA). Resultant CDK4^R24C^ cDNA was transferred into the pCDH-CMV-MCS-EF1-Hygro vector (System Biosciences, Mountain View, CA), which carries the hygromycin phosphotransferase (HPT) gene, resulting in pCDH-CMV-CDK4^R24C^-EF1-Hygro. Similarly, cDNA encoding cyclin D1 was PCR amplified and inserted into pCDH-CMV-MCS-EF1-Bsd, which was constructed by replacing the puromycin *N*-acetyltransferase gene in pCDH-CMV-MCS-EF1-Puro (System Biosciences) with the blasticidin S deaminase (BSD) gene, resulting in pCDH-CMV-cyclin D1-EF1-Bsd. pLOX-TERT-IRES-TK (Addgene plasmid #12,245), a lentiviral vector harboring cDNA encoding human TERT was a gift from Dr. Didier Trono. Human embryonic kidney (HEK) 293 T cells were cultured in three 10-cm dishes, and cells in each dish were transfected with one of the above three lentiviral vectors plus psPAX2 (Addgene plasmid #12,260, a gift from Dr. Didier Trono) and pCMV-VSV-G (Addgene plasmid #8454, a gift from Dr. Bob Weinberg) [11] at a ratio of 3:2:1 using the calcium phosphate–DNA co-precipitation method [12]. Forty-eight hours later, conditioned media containing infectious recombinant lentiviruses were recovered.

### Isolation of carcinoma cells and gene transfer

Fragments of tumor tissue obtained at surgery were cut into small pieces, suspended in 10 mL of 1 mg/mL Collagenase/Dispase (Roche Diagnostics, Mannheim, Germany) and incubated at 37 °C for 30 min [13]. To remove undigested tissue fragments, the slurry was passed through a 40-μm pore cell strainer. After washing with phosphate-buffered saline (PBS), cells were cultured in RPMI 1640 medium supplemented with 10% fetal bovine serum (FBS), 100 U/mL penicillin, 100 μg/mL streptomycin and 0.25 μg/mL amphotericin B (Nacalai Tesque, Kyoto, Japan). Twenty-four hours later, cells were infected with the above three recombinant lentiviruses by replacing the regular medium with a mixture of the three conditioned media prepared above. After another 24 h, virus-containing medium was changed to regular medium supplemented with 400 μg/mL of hygromycin B and 10 μg/mL of blasticidin S (Thermo Fisher Scientific) to select transformed cells. Growth and morphology of cultured cells were observed with an inverted phase-contrast microscope IX71 (Olympus, Tokyo, Japan).

### Confirmation of transgene incorporation

Incorporation of the three exogenous genes mentioned above into the host cell genome was confirmed by reverse transcription-PCR (RT-PCR), as described [14] using the following primer pairs: 5’-ATgTTCggggATTCCCAATACgAg-3’ and 5’-CTATCggCgAgTACTTCTACACAg-3’ for HPT in the plasmid harboring CDK4^R24C^; 5’-gCTACAATCAACAgCATCCCCATC-3’ and 5’-CACATAACCAgAgggCAgCAATTC-3’ for BSD in the plasmid harboring cyclin D1; and 5’-gTCTTCTTgACgAgCATTCCTAgg-3’ and 5’-ACATgTAAAgCATgTgCACCgAgg-3’ for the internal ribosome entry site (IRES) in the plasmid harboring TERT. As a control, endogenous glyceraldehyde-3-phosphate dehydrogenase (GAPDH) expression was detected using the primer pair 5’-ATggggAAggTgAAggTCggAgTC-3’ and 5’-CAgAgATgATgACCCTTTTggCTC-3’.

### Transmission electron microscopy

Electron microscopy specimens were prepared as previously described [15] and observed with an H-7650 transmission electron microscope (Hitachi, Tokyo, Japan).

### Growth curve analysis and doubling time

Cells were seeded into wells of six-well plates at a concentration of 1.0 × 10^5^ cells/well. Cells in wells were counted in triplicate at 48-h intervals over a 16-day period. Doubling times were calculated using a web-based doubling-time calculator (https://www.doubling-time.com/compute.php).

### Cell cycle analysis

Trypsinized monodispersed cells were fixed in ice-cold 70% ethanol at 4 °C for 2 h. Cells were then re-suspended in 0.5 mL PBS, and 5 μL Cell Cycle Assay Solution Blue (Dojindo Laboratories, Mashiki, Japan) was added and incubated for 15 min at 37 °C under light-shielded conditions. Stained nuclei were analyzed using FACSCanto II (BD Biosciences, San Jose, CA) with FlowJo software (Tree Star, Ashland, OR).

### Karyotyping

Chromosomal G-band analysis was performed as described previously [15]. Karyotypes were described according to the International System for Human Cytogenomic Nomenclature (ISCN) 2020 [16].

### Sanger sequencing

Mutations in DNA polymerase ε (*POLE*) were detected by Sanger sequencing as described [15]. Briefly, total RNA was extracted from cells using ISOGEN reagent (Nippon Gene, Tokyo, Japan), and single-stranded cDNA was synthesized as described [17]. DNA fragments corresponding to amino acid residues 270–475 of POLE, the hotspot region of the exonuclease domain [18], were amplified by PCR and the products were purified using QIAquick® Gel Extraction Kit (QIAGEN, Venlo, The Netherlands). Sequencing reactions were carried out using a BigDye® Terminator v.1.1 Cycle Sequencing Kit (Thermo Fisher Scientific) and sequenced using an Applied Biosystems 3500 Genetic Analyzer (Thermo Fisher Scientific).

### Short tandem repeat (STR) analysis

Genomic DNA was extracted from cells using a NucleoSpin® Tissue (Takara Bio), and 16 STR loci were detected by multiplex PCR using a PowerPlex® 16 HS System (Promega, Madison, WI). STR profiles were compared with those recorded in the Expasy Profile Database (https://www.cellosaurus.org/str-search/), as described [15].

### Monoclonal antibodies

The following monoclonal antibodies (mouse IgG unless otherwise noted) served as primary antibodies: OV-TL 12/30 (Dako, Glostrup, Denmark), recognizing cytokeratin 7 (CK7); Ks20.8 (Dako), recognizing CK20; BC12 (Nichirei Biosciences, Tokyo, Japan), recognizing paired box 8 (PAX8); V9 (Dako), recognizing vimentin; WT49 (Leica Biosystems, Newcastle Upon Tyne, UK), recognizing Wilms tumor 1 (WT1); 1D5 (Dako), recognizing estrogen receptor (ER); 1A6 (Dako), recognizing progesterone receptor (PgR); DO-7 (Nichirei Biosciences), recognizing p53; SP218 (rabbit IgG; Abcam, Cambridge, UK), recognizing phosphatase and tensin homolog deleted on chromosome 10 (PTEN); ES05 (Leica Biosystems), recognizing mutL homolog 1 (MLH1); 79H11 (Leica Biosystems), recognizing mutS homolog 2 (MSH2); EP49 (rabbit IgG; Leica Biosystems), recognizing mutS homolog 6 (MSH6); and EP51 (rabbit IgG; Leica Biosystems), recognizing postmeiotic segregation increased 2 (PMS2).

### Histological and immunohistochemical analyses

Formalin-fixed, paraffin-embedded tissue sections were stained with hematoxylin and eosin (H&E) or immunostained with the monoclonal antibodies noted above. Immunohistochemical staining was performed using the Histofine system (Nichirei Biosciences), according to the manufacturer’s protocol [19].

### Immunofluorescence staining

Immunofluorescence staining of JFE-21 cells was performed as previously described [20, 21].

### Anticancer drugs

Paclitaxel (catalog number Y0000698) and carboplatin (catalog number BP711) were purchased from Merck KGaA (Marmstadt, Germany). Paclitaxel was first dissolved in 100% ethanol to make a 10 mM solution, and then diluted 1:100 with sterile water to make a 100 μM stock solution. Carboplatin, on the other hand, was directly dissolved in sterile water to prepare a 50 mM stock solution. These stock solutions were further diluted with phenol red-free RPMI 1640 medium to the desired final concentration.

### Cell viability assay

One hundred μL of phenol red-free RPMI 1640 medium containing 2 × 10^4^ cells was dispensed into each well of a 96-well culture plate and cultured overnight. Then, 100 μL of medium containing various concentrations of paclitaxel or carboplatin was added to the wells and further cultured. Seventy-two hours later, 20 μL of substrate solution containing 5 mM 2-(4-iodophenyl)−3-(4-nitrophenyl)−5-(2,4-disulfophenyl)−2*H*-tetrazolium (Dojindo Laboratories, Mashiki, Japan) and 0.2 mM 1-methoxy-5-methylphenazinium methylsulfate (Dojindo Laboratories) was added to the wells and incubated at 37 °C in a humidified atmosphere with 5% CO_2_. Four hours later, absorbance of each well was measured at 450 nm using a SpectraMax iD3 microplate reader (Molecular Device, Sunnyvale, CA). The percentage of viable cells was calculated by dividing the absorbance of treated wells by that of untreated wells. The half maximal inhibitory concentration (IC_50_) was calculated using GraphPad Prism 7 software (GraphPad Software, San Diego, CA).

## Results

### Pathological diagnosis of the patient’s tumor tissue

**Fig. 1:**
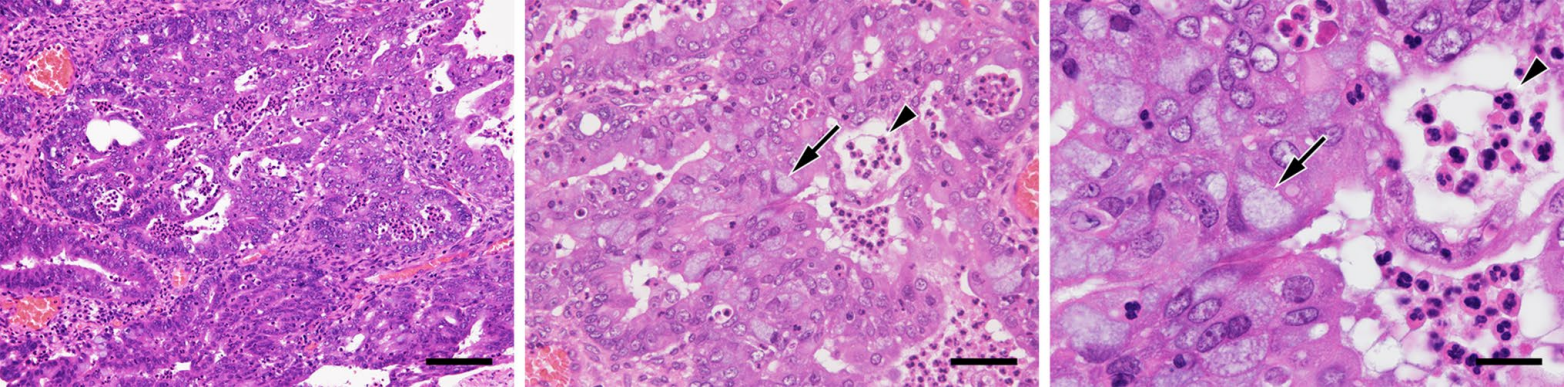
Histological appearance of the patient’s ovarian tumor. Tissue sections were stained with hematoxylin and eosin (H&E). Arrows, mucinous differentiation of tumor epithelial cells; arrowheads, aggregates of neutrophils in tumor glands. Bars = 100 μm, left panel; 50 μm, middle panel; and 20 μm, right panel

**Fig. 2:**
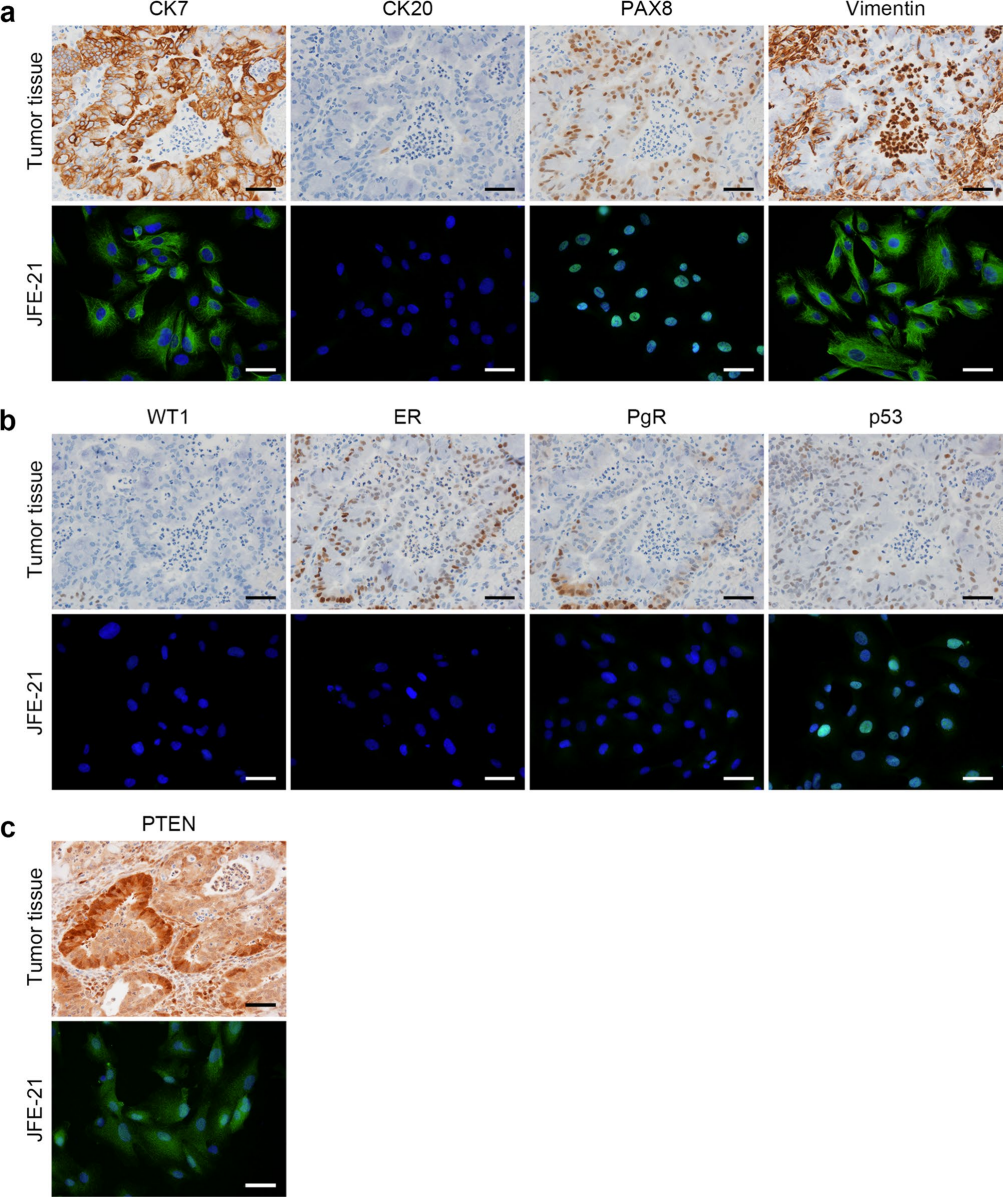
**a**, **b**, **c** Expression profiles of indicated ovarian carcinoma markers in the patient’s tumor tissue and in JFE-21 cells. Tissue sections (upper panels in **a**, **b**, and **c**) or cells on coverslips (lower panels in **a**, **b**, and **c**) were immunostained for indicated markers. Immunohistochemistry signals were visualized with 3,3’-diaminobenzidine (DAB; brown), and tissues were counterstained with hematoxylin. Immunofluorescence signals were derived from Alexa Fluor 488 on secondary antibodies and nuclei were marked with 4′6-diamidino-2-phenylindole (DAPI; blue). Note that both neoplastic epithelial cells and neutrophils in tumor glands are vimentin positive (a, upper far right panel). Bar = 50 μm

Histologically, the tumor consisted of a collection of atypical endometrioid glands arranged back to back or exhibiting a cribriform pattern (Fig. 1, left panel). These glands had smooth luminal surfaces and often showed mucinous differentiation (arrows) and neutrophil accumulation in the lumen (arrowheads). Mitotic figures were scarcely observed. Immunohistochemically, as shown in Fig. 2a (upper panels), tumor cells were CK7 positive, CK20 negative and PAX8 positive, a pattern consistent with carcinomas arising from the ovary. Cells were also positive for vimentin, which is usually expressed in endometrioid but not mucinous carcinoma [22]. In addition, as shown in Fig. 2b (upper panels), carcinoma cells were negative for WT1, a reliable marker of serous carcinoma, and positive for ER and PgR, which often mark endometrioid carcinoma [23]. The p53 expression pattern was wild type, and loss of PTEN expression was not observed (Fig. 2c, upper panel). Overall, these histological features and immunohistochemical profiles best fit with a pathological diagnosis of ovarian endometrioid carcinoma.

### Cytological characteristics of JFE-21 cells

**Fig. 3:**
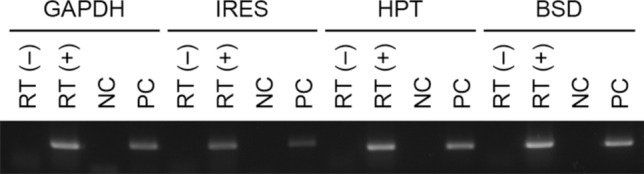
Gene expression analysis of JFE-21 cells by reverse transcription-polymerase chain reaction (RT-PCR). Misexpression of telomerase reverse transcriptase (TERT), mutant cyclin-dependent kinase 4 (R24C) (CDK4^R24C^) and cyclin D1 genes was confirmed by detection of transcripts corresponding to the internal ribosome entry site (IRES), the hygromycin phosphotransferase (HPT) gene and the blasticidin S deaminase (BSD) gene, respectively. RNA samples were treated with (+) or without (−) reverse transcriptase (RT). NC, negative control (distilled water); PC, positive control (plasmid harboring target cDNA). GAPDH, glyceraldehyde-3-phosphate dehydrogenase

**Fig. 4:**
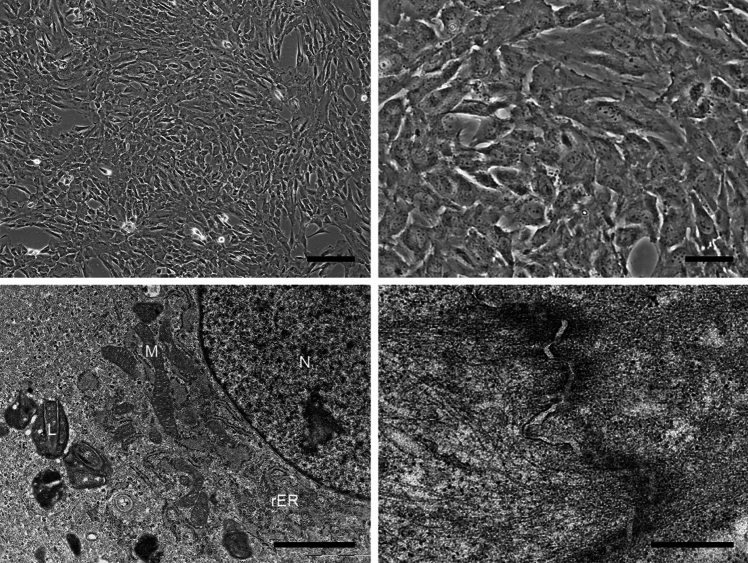
Morphological characteristics of JFE-21 cells. Phase-contrast micrograph (upper panels) and transmission electron micrograph (lower panels) of JFE-21 cells. L, lysosomes, M, mitochondria; N, nuclei; and rER, rough endoplasmic reticulum. Bars = 200 μm, upper left panel; 50 μm, upper right panel; 2 μm, lower left panel; and 500 nm, lower right panel

**Fig. 5:**
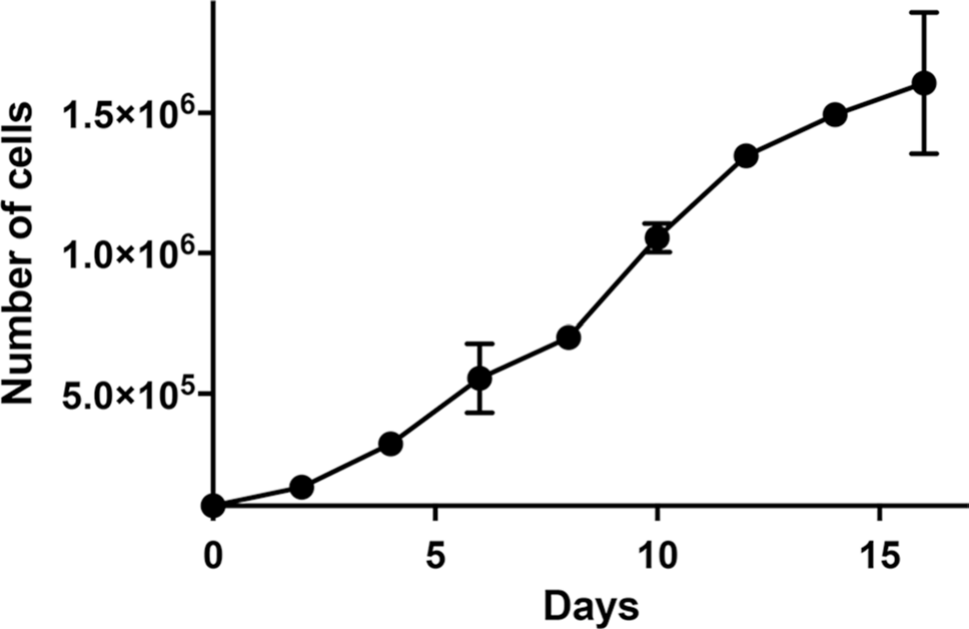
Growth curve of JFE-21 cells. Data are presented as means ± standard error of the mean (SEM) of triplicate measures

**Fig. 6:**
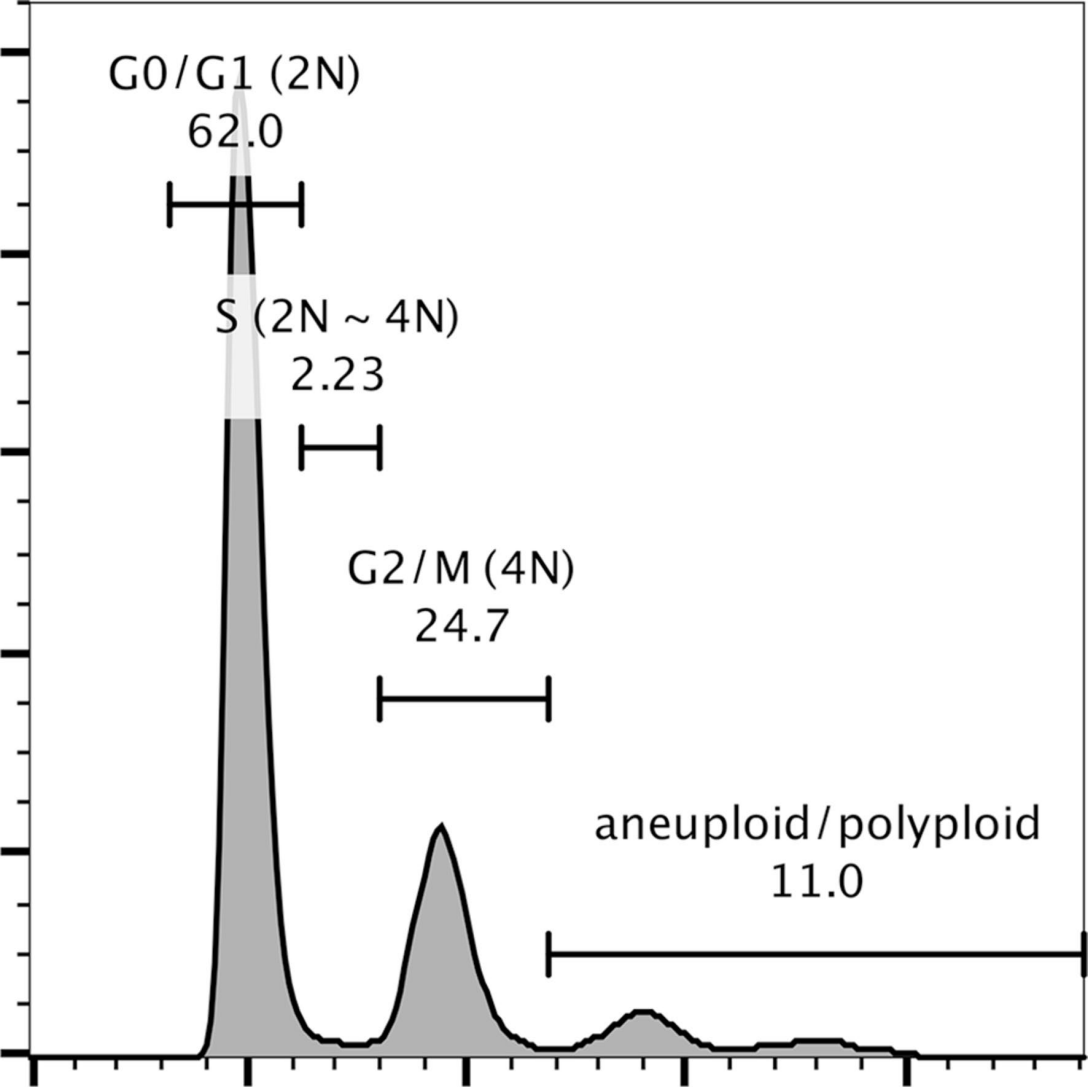
Flow cytometry histograms showing population distribution relative to DNA content. Numbers indicate percentages of cell populations

**Fig. 7:**
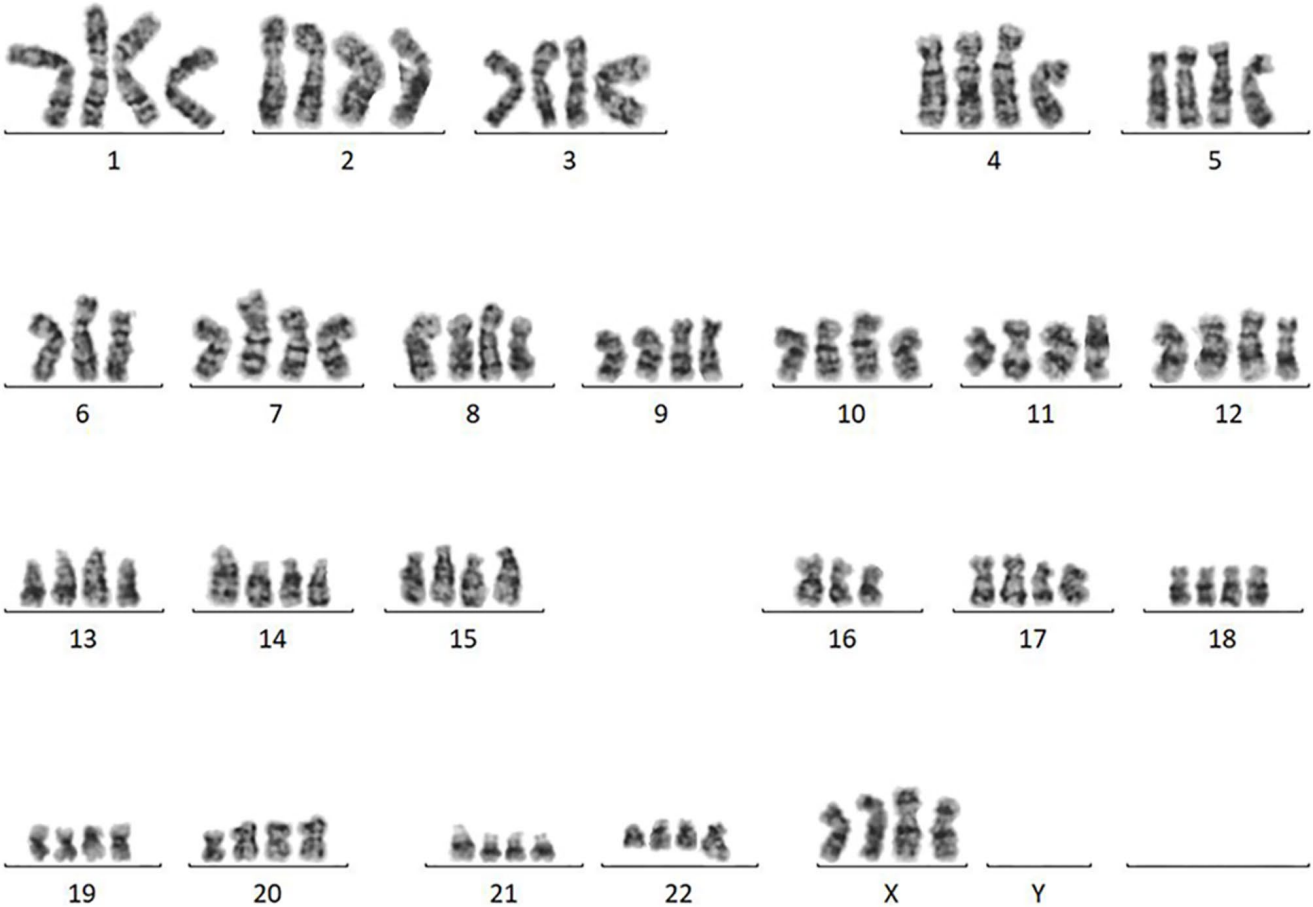
Representative Giemsa-banded karyotype of JFE-21 cells showing near tetraploidy (4N ±)

We then infected cultures of the patient’s tumor cells with recombinant lentiviruses carrying the CDK4^R24C^, cyclin D1 and TERT genes, and continued to grow them under standard cell culture conditions. Two weeks later, we observed a colony of epithelial cells from which the present cell line, designated JFE-21, was cloned using stainless steel cloning cylinder and trypsin. RT-PCR analysis demonstrated that indeed, JFE-21 cells constitutively expressed exogenous CDK4^R24C^, cyclin D1 and TERT genes (Fig. 3). At the time of this writing, these cells have been proliferating for more than 6 months and have been passaged over 25 times. As shown in Fig. 4 (upper panels), phase-contrast microscopy indicated that the cells grew without contact inhibition and formed a jigsaw puzzle-like monolayer. Cells were polygonal in shape and exhibited large round or oval nuclei. As shown in Fig. 4 (lower panels), transmission electron microscopy analysis showed that JFE-21 cells had well-developed rough endoplasmic reticulum (rER), mitochondria and lysosomes in their cytoplasm (left panel) and contacted neighboring cells via desmosomes (right panel). The proliferation characteristics of JFE-21 cells fit a logistic curve (Fig. 5), and at log phase the doubling time was calculated to be 55.8 ± 7.0 h. Cell cycle analysis revealed percentages of cells in various phases to be: 62.0% in G_0_/G_1_ (2N), 2.23% in S (between 2 and 4N) and 24.7% in G_2_/M (4N), with the remaining 11.0% aneuploid or polyploid (Fig. 6). Accordingly, G-band karyotyping of JFE-21 cells revealed the following near-tetraploid (4N ±) karyotype: 88 ~ 91,XXXX,–6[cp4]/45,XX,–6[4]/46,XX[22] (Fig. 7). Finally, STR analysis revealed that the profiles of JFE-21 cells (Table 1) did not match those of any existing cell lines deposited in public banks, indicating that JFE-21 is unique and not cross-contaminated or misidentified.

**Table 1:** STR genotyping of JFE-21 cells

Microsatellite (chromosome)	JFE-21
D3S1358	14, 17
TH01	6, 9
D21S11	29, 30
D18S51	15, 16
Penta_E	5, 15
D5S818	9, 11
D13S317	10
D7S820	11, 12
D16S539	10, 11
CSF1PO	11, 12
Penta_D	9
AMEL	X
vWA	17, 18
D8S1179	10, 15
D8S1179	8, 11
FGA	21, 23

### Expression profiles of ovarian carcinoma markers in JFE-21 cells

We then carried out immunofluorescence staining of JFE-21 cells to assess the expression of a series of ovarian carcinoma markers. As shown in Fig. 2 (lower panels), JFE-21 cells were CK7 positive, CK20 negative and PAX8 positive, a pattern identical to the patient’s tumor tissue and consistent with carcinomas arising from the ovary. Moreover, JFE-21 cells were vimentin positive, WT1 negative and showed a wild-type p53 expression pattern and no loss of PTEN expression, a pattern also identical to the patient’s tumor tissue and consistent with endometrioid carcinoma. On the other hand, the cells were negative for both ER and PgR, in contrast with the patient’s tumor tissue, which was positive for both.

### JFE-21 cells are classified as the NSMP subtype of ovarian endometrioid carcinoma

**Fig. 8:**
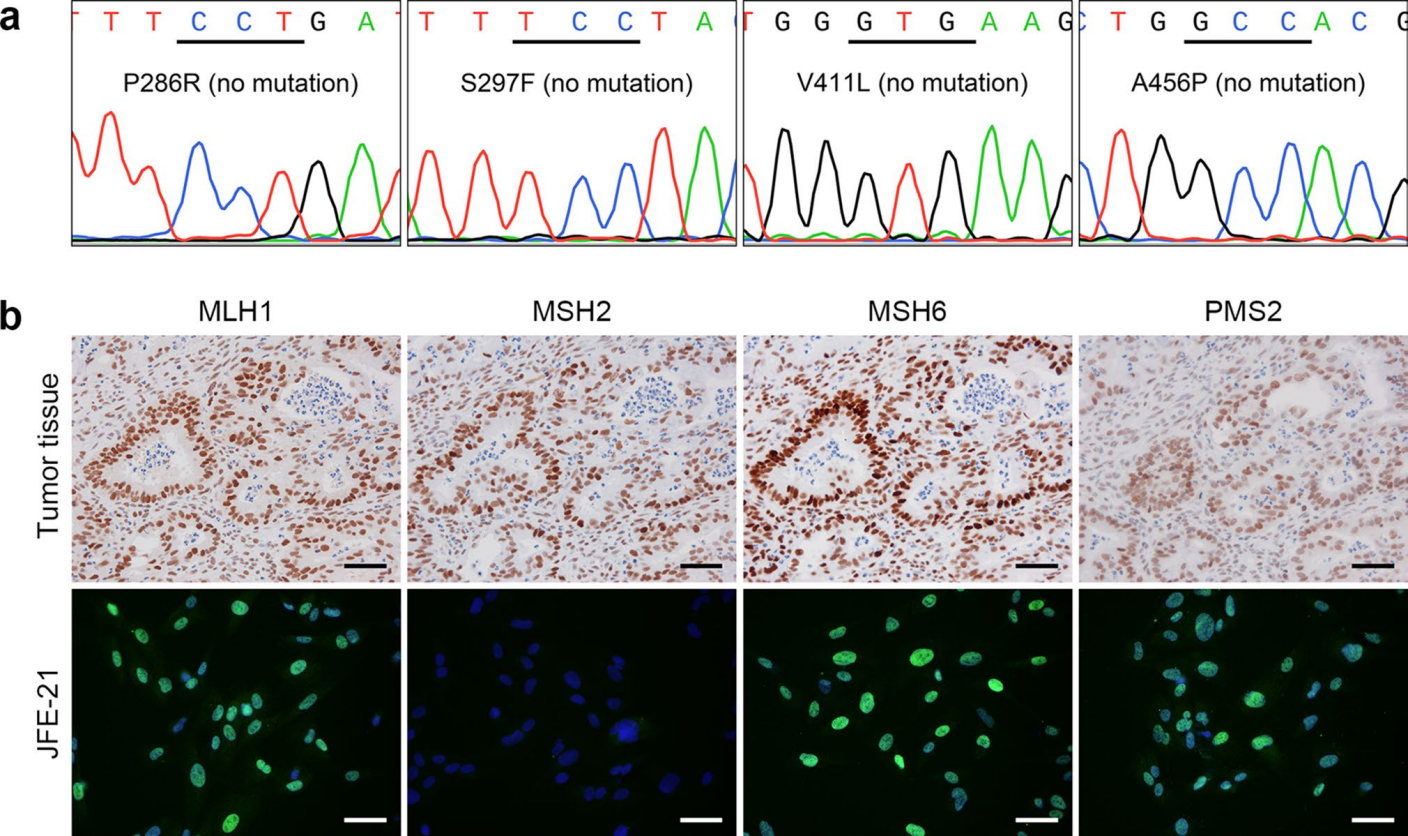
**a** Sanger sequencing chromatograms of four representative *POLE* mutation sites in JFE-21 cells. Note that JFE-21 cells do not harbor these mutations. **b** Expression profiles of indicated mismatch repair (MMR) proteins in the patient’s tumor tissue and in JFE-21 cells. Tissue sections (upper panels) or cells on coverslips (lower panels) were immunostained for indicated MMR proteins. Immunohistochemistry signals were visualized with 3,3’-diaminobenzidine (DAB; brown), and tissues were counterstained with hematoxylin. Immunofluorescence signals were derived from Alexa Fluor 488 on secondary antibodies and nuclei were marked with 4′6-diamidino-2-phenylindole (DAPI; blue). Bar = 50 μm

Similar to the molecular subtypes of endometrial endometrioid carcinoma defined by The Cancer Genome Atlas [24], four molecular subtypes of ovarian endometrioid carcinoma have been proposed: *POLE* mutant, mismatch repair (MMR) deficient, p53 abnormal, and no specific molecular profile (NSMP) [25]. To determine which subtype the JFE-21 cells belong to, we performed Sanger sequencing of DNA fragments corresponding to amino acid residues 270–475 of POLE, the hotspot region of the exonuclease domain with known recurrent mutations including P286R, S297F, V411L and A456P [26]. The results showed that JFE-21 cells did not harbor any of the recurrent mutations reported to date (Fig. 8a). We also performed immunohistochemical analysis on four MMR proteins, namely MLH1, MSH2, MSH6 and PMS2, and found that all four MMR proteins were expressed in patient tumor tissue (Fig. 8b, upper panels). Interestingly, MSH2 expression was not detected in JFE-21 cells (Fig. 8b, lower panels), while the reason for this is unclear. As already mentioned, the expression pattern of p53 was wild type (see Fig. 2b, right panels). Based on these findings, JFE-21 cells can be classified as the NSMP subtype.

### JFE-21 cells are sensitive to both paclitaxel and carboplatin

**Fig. 9:**
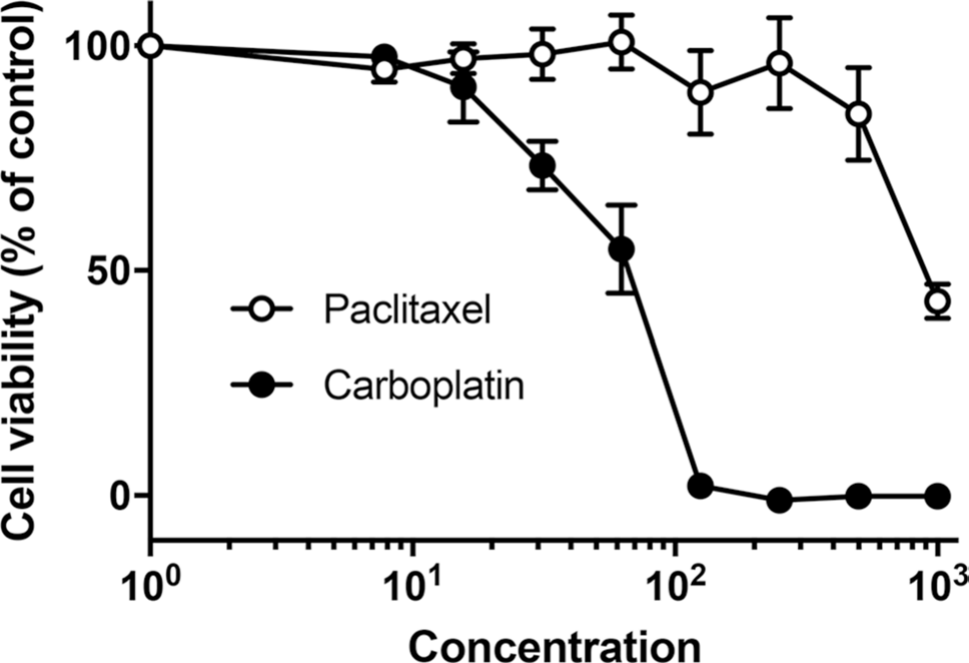
Sensitivity of JFE-21 cells to paclitaxel (white circles) and carboplatin (black circles) as evaluated by cell viability assay. Data are presented as means ± standard error of the mean (SEM) of quadruplicate measurements. Concentration units (x-axis) are nM for paclitaxel and μM for carboplatin

We then performed a cell viability assay in the presence or absence of a series of concentrations of paclitaxel or carboplatin administered to the patient as treatment. As shown in Fig. 9, JFE-21 cells were sensitive to both anticancer drugs, with an IC_50_ of ~ 900 nM for paclitaxel and 57.16 μM for carboplatin.

## Discussion

In the present study, we established the human ovarian endometrioid carcinoma cell line JFE-21 by constitutive expression of exogenous CDK4^R24C^, cyclin D1 and TERT genes. That line has proliferated continuously for over 6 months, with a doubling time of ~ 55 h, and exhibits expression profiles of a series of ovarian carcinoma markers consistent with the NSMP subtype of ovarian endometrioid carcinoma. Thus, this immortalization strategy can be an option for establishing cell lines from low-grade cancers, including ovarian endometrioid carcinoma.

In our previous attempts to establish ovarian lines, cells derived from patients’ ovarian cancer tissues could be cultured for only a limited period of time (approximately 2–3 weeks) and most did not proliferate beyond that. This challenge indicates that even cancer cells that proliferate autonomously in the human body undergo premature cell cycle arrest in a cell culture environment [2]. To overcome this hurdle, we employed a cell immortalization strategy consisting of i) inactivation of the p16^INK4a^-pRb pathway and ii) acquisition of TERT activity [27], which has been successfully used to immortalize human myogenic cells [8] and hepatocytes [9].

For successful cell immortalization, two principal senescence barriers must be overcome: one is replicative senescence and the other is stress-induced senescence. Expression of exogenous TERT in cells extends their replicative capacity and, in some cases, immortalizes cells [28, 29]. However, stress-induced senescence is independent of telomere length [30]. Thus, reliable immortalization of cells usually requires TERT activity coupled with inactivation of the p16^INK4a^–pRb pathway. As mentioned above, stress-induced premature senescence due to activation of p16^INK4a^–pRb pathway often hampers establishment of cell lines. In such a situation, it would be beneficial to introduce exogenous CDK4^R24C^ and cyclin D1 genes in addition to TERT gene, as shown in this study.

RT-PCR analysis revealed that JFE-21 cells express all three exogenous genes, namely CDK4^R24C^, cyclin D1 and TERT. To establish that line, we transfected cells using lentiviral vectors, which may have resulted in high transfection efficiency. Furthermore, lentiviral vectors harboring CDK^R24C^ and cyclin D1 contained hygromycin- and blasticidin-resistance genes, respectively, allowing us to select transduced cells in the appropriate antibiotics. Unfortunately, the lentiviral vector harboring TERT did not contain a drug-resistance gene, but rather harbored the thymidine kinase (TK) gene, which could not be used for positive selection. For future studies, it would be beneficial in selecting transgenic cells if the TK gene in the TERT vector was replaced with a drug-resistance gene such as puromycin *N*-acetyltransferase gene.

Some ovarian cancer cell lines reported to date have not been properly annotated [31], which is a major problem because, once in culture, these cells no longer possess easily identifiable morphological features that aid in histological classification [31]. Kalloger et al. developed the Calculator for Ovarian Subtype Prediction (COSP), which combines nine immunohistochemical markers with a prediction algorithm [32]. Using COSP, Anglesio et al. validated 32 ovarian cancer cell lines and predicted their histological types. As a result, A2780 cells, which were previously reported to be derived from adenocarcinoma, as well as ES-2 cells, which were thought to be derived from clear cell carcinoma, were both predicted to be of endometrioid carcinoma origin with high probability [31]. However, neither line shows expression profiles of ovarian carcinoma markers consistent with ovarian endometrioid carcinoma: A2780 cells do not express CK7 and ER and only weakly express PAX8, and ES-2 cells do not express PAX8 or ER but weakly express CK7 [2]. The same authors also showed that TOV-112D cells, a line reported to originate from ovarian endometrioid carcinoma, express neither CK7, PAX8 nor ER [2]. Given these observations, the JFE-21 cells established here may be a more representative ovarian endometrioid carcinoma line than other reported cell lines.

Despite these findings, the line was negative for the hormone receptors ER and PgR, even though the patient’s original cancer tissue contained a mixture of cells both positive and negative for both hormone receptors (see Fig. 2b). Reasons underlying this phenomenon are not fully understood, but it is presumably due to in vitro selection of receptor-negative cells [3]. Thus, a clone of JFE-21 negative for both ER and PgR may have exhibited a growth advantage, leading to their selection.

Finally, we show in this study that JFE-21 cells are sensitive to paclitaxel and carboplatin administered to the donor as therapy. This data is one piece of evidence that this cell line reflects in vivo conditions and is a valid model of patient-derived ovarian endometrioid carcinoma. Thus, JFE-21 cells could be used for cancer research to better understand the pathophysiology of this form of ovarian carcinoma and for validation of new diagnostic and therapeutic approaches for this histological type of cancer.

## Data Availability

Data supporting findings reported in this study are available from the corresponding author upon reasonable request.
